# Identification of C-terminal Hsp70-interacting protein as a mediator of tumour necrosis factor action in osteoblast differentiation by targeting osterix for degradation

**DOI:** 10.1111/jcmm.12553

**Published:** 2015-03-26

**Authors:** Jianmin Xie, Jieruo Gu

**Affiliations:** a Department of Rheumatology, The Second Affiliated Hospital of Nanjing Medical UniversityNanjing, China; b Department of Rheumatology, The Third Affiliated Hospital of Sun Yat-sen UniversityGuangzhou, China

**Keywords:** TNF-α, osterix, osteoblast, CHIP

## Abstract

In patients with inflammatory arthritis, tumour necrosis factor (TNF)-α are overproduced in inflamed joints. This leads to local erosion of cartilage and bone, periarticular osteopenia, as well as osteoporosis. But less is known regarding the molecular mechanisms that mediate the effect of TNF-α on osteoblast function. The purpose of this study was to test that C terminus of Hsc70-interacting protein (CHIP) has a specific role in suppressing the osteogenic activity of osteoblasts under inflammatory conditions. C2C12, MC3T3-E1 and HEK293T cell lines were cultured and cotransfected with related plasmids. After transfection, the cells were cultured further in the presence or absence of murine TNF-α and subjected to real time RT-PCR, Western blot, Ubiquitination assay, Co-immunoprecipitation, Luciferase reporter assay, Small interfering RNAs and Mineralization assay. The expression levels of TNF-α-induced CHIP and Osx were examined by RT-PCR and Western blot analysis. Co-immunoprecipitation and ubiquitination assays revealed ubiquitinated Osx, confirmed that CHIP indeed interacted with Osx and identified K55 and K386 residues as the ubiquitination sites in Osx, Luciferase reporter assay and Small interfering RNAs examined whether TNF-α target the bone morphogenetic protein signalling through CHIP. We established stable cell lines with the overexpression of HA-CHIP, Mineralization assay and CHIP siRNA demonstrated the important roles of CHIP on osteoblast function in conditions in which TNF-α is overexpressed. We found that the K55 and K386 residues are ubiquitination site(s) in Osx, and that TNF-α inhibits osteoblast differentiation by promoting Osx degradation through up-regulation of E3 ubiquitin ligase CHIP in osteoblast. Thus, CHIP targets Osx for ubiquitination and degradation in osteoblasts after chronic exposure to TNF-α, and inhibition of CHIP expression in osteoblasts may be a new mechanism to limit inflammation-mediated osteoporosis by promoting their differentiation into osteoblasts.

## Introduction

Bone is a major target of many inflammatory rheumatic diseases, including rheumatoid arthritis (RA) and ankylosing spondylitis (AS) 1,2. Inflammation leads to a wide range of changes in osteoblast differentiation. Osteoblast differentiation activity is dependent on a strict coupling mechanism of osteoclast resorption and new matrix deposition by osteoblasts, and an imbalance between these two activities leads to pathological states such as osteoporosis. Osteoporosis and fragility fractures are common and preventable complications of RA and AS 3. Tumour necrosis factor (TNF-α) and other cytokines are overproduced in the inflamed joints of RA and AS patients as a result of various cells infiltrating the synovial membrane, which leads to severe local erosion of the cartilage and bone, periarticular osteopenia and systemic osteoporosis. Tumour necrosis factor-α is a pleotropic cytokine known to be involved in the progression of several pro-inflammatory disorders. Transcription factor Snail regulates TNF-α-mediated synovial fibroblasts activation in rheumatoid joint. Many therapeutic agents have been designed to counteract the effect of TNF in RA as well as a number of cancers 4,5.

Little is known regarding the role of TNF-α in osteoblast biology. The inhibitory effects of TNF-α on osteoblast differentiation *in vitro* were first described in neonatal rat calvarial organ cultures in 1987 6. Subsequent studies demonstrated that TNF-α inhibits the recruitment of osteoblast progenitors, reduces the expression of genes produced by mature osteoblasts, and promotes osteoblast apoptosis through the nuclear factor-ĸB signalling pathway 7–11. Every step in the commitment of an osteoblast precursor is orchestrated by the expression of skeletal-specific transcription factors 12–14, and those that are critical for osteoblast differentiation include runt-related factor (Runx2) and osterix (Osx) because their deletion results in a cartilaginous skeleton 15,16. Previous studies reported that TNF-α is a potent inhibitor of the skeletal transcription factors Runx2 17,18. The TNF-α-induced reduction in the nuclear Runx2 protein was greater than expected compared with the decrease in total Runx2 mRNA. Furthermore, pharmacological inhibitors of the cell survival-promoting kinases fail to reverse the inhibitory effects of TNF-α on osteoblast differentiation *in vitro* 19,20, suggesting that other signal pathways may be involved.

Osx has been reported to be required for bone morphogenetic protein-2 (BMP-2) and Runx2-induced osteoblast differentiation and bone growth in post-natal and adult mice 21,22, to be a more restricted transcription factor than Runx2 in osteoblast differentiation and to act downstream of Runx2 23. Osx is specifically expressed in osteoblasts, and the expression of Osx becomes stronger as osteoblast differentiation occurs. We and others have been suggested that the inhibition of Osx expression in inflammatory arthritis may impair osteoblast differentiation and predispose the patient to increased periarticular and systemic bone loss 24–26^.^ At present, the regulation of Osx has not been fully investigated, and less is known regarding its post-translational regulation.

Recently, the ubiquitin–proteasome system has been implicated in the regulation of the BMP-2 and transforming growth factor-β signalling pathways in various cell types. Others have demonstrated that the E3 ubiquitin ligase, C terminus of Hsc70-interacting protein (CHIP), regulates osteoblast differentiation by promoting the proteasomal degradation of the BMP signalling proteins Smad1 and Smad5 and of the osteoblast transcription factor Runx2 27,28. CHIP was originally identified as a cochaperone involved in protein folding. In addition, CHIP has Ubox-dependent ubiquitin ligase activity and triggers the degradation of chaperone client proteins 29,30. Osteoblast activity is augmented in ubiquitin ligase-deficient mice that generate adult onset osteosclerosis with increased bone mass 31.

On the basis of the findings from these studies, we tested the hypothesis that TNF-α inhibits osteoblastic function by up-regulating CHIP E3 ligases that trigger degradation of the Osx protein. Thus, our results shed some light on a novel molecular mechanism for the inhibition of osteoblasts by TNF-α that involves the post-transcriptional regulation of protein function through CHIP E3 ligase-mediated proteasomal degradation, providing further elucidation of the regulation of bone metabolism.

## Materials and methods

### Antibodies

Anti-Flag, anti-Myc and anti-β-actin were purchased from Sigma-Aldrich (St. Louis, MO, USA),. The anti-Osx monoclonal antibody was purchased from MBL (Woburn, MA, USA). The anti-ubiquitin and anti-HA monoclonal antibodies and anti-CHIP polyclonal antibody were purchased from Santa Cruz Biotechnology (Santa Cruz, CA, USA).

### Plasmids, cell culture, transfection and co-immunoprecipitation

pCMV-Flag-Osx, pCMV-Myc-CHIP and pCDNA-HA-CHIP were generated according to standard molecular techniques and subjected to DNA sequencing. A luciferase reporter construct containing the mouse osteocalcin (Ocn) promoter (Ocn-Luc) was created using the pGL3-basic vector (Promega, Madison, WI, USA). The Ocn promoter fragment was determined by sequencing. Lys (K) to Arg (R) mutants of Osx were generated using the Quickchange site-directed mutagenesis kit (Stratagene). The mutated fragment was confirmed by sequencing (Sigma-Aldrich). HEK293T and C2C12 cells were cultured in DME supplemented with 10% FBS (HyClone, Rockford, IL, USA). MC3T3-E1 cells were cultured in α-MEM supplemented with 10% FBS. The cells were cotransfected with FLAG-tagged-Osx and Myc-tagged-CHIP or HA-tagged-CHIP using Tfx-20 (Promega) in a 100-mm dish. Empty vector was added to balance the total DNA amounts. After transfection (24 hrs), the cells were cultured further in the presence or absence of murine TNF-α (R&D Systems) and subjected to reverse transcription (RT)-PCR (Promega) or western blot analysis. At 48 hrs, the cells lysates were incubated. Co-immunoprecipitation was performed according to the manufacturer’s instructions. To inhibit proteasome degradation, the cells were incubated with 50 μM MG132 (Sigma-Aldrich) for 4 hrs. These studies were carried out in cell lines and no institutional ethics approval or patient consent was required. Caspase-3 activity and cell viability was assessed *via* the MTT assay as described previously 32.

### Ubiquitination assay

In 293T cells, HA-tagged ubiquitin was cotransfected with Flag-Osx and Myc-CHIP in the presence of 50 μM MG132 for 4 hrs before being harvested. MC3T3-E1 cells were treated with 10 ng/ml TNF-α for 48 hrs in the presence of PBS or 50 μM MG132 for the last 4 hrs of TNF-α treatment. The cell lysates were then incubated with an anti-Flag antibody and protein G agarose (Sigma-Aldrich) overnight at 4°C. Ubiquitination assays were performed as described previously 33.

### Small interfering RNAs

The small interfering RNAs (siRNAs) targeting the CHIP messenger RNA (mRNA) were designed using the Ambion website. Lentivirus without the transgene was used as the negative control. The siRNAs were delivered using lentivirus particles (1 ml, 108 TU/μl) by Gene Pharma (Shanghai Gene Pharma Co, Ltd., Shanghai, China). The CHIP siRNA (LeshCHIP) sequence was 5-AACAGGCACTTGCTGACTG-3. Transfected MC3T3-E1 cells were stably selected using G418 (600 ng/ml) and pooled for further experiments. Two days after transfection, the cells were harvested for quantitative real-time RT-PCR or western blot analysis. The experiments were repeated three times with similar results.

### Western blotting and Luciferase assay

The cells were harvested in direct lysis buffer. Protein extraction and immunobloting were performed according to the manufacturer’s instructions. MC3T3-E1 cells were transiently transfected with the BRE-Luc BMP signalling reporter, CHIP siRNA vectors or empty vector, which was used to equalize the total DNA amount. The cells were treated with 10 ng/ml TNF-α for 48 hrs, followed by a 20-hr incubation in the presence or absence of 100 ng/ml BMP-2 (R&D Systems). HEK 293T cells were cotransfected with the WT Flag-Osx plasmid or Lys-to-Arg mutants and the Ocn-Luc and *Renilla* luciferase reporter vectors, and luciferase activity was determined after 24 hrs of transfection. C2C12 cells were cotransfected with Flag-Osx (WT) or the K55R or K386R mutants and the Ocn-Luc reporter, *Renilla* luciferase reporter vector and CHIP siRNA construct. The cells were then treated with 10 ng/ml TNF-α for 48 hrs, followed by a 20 hrs incubation in the presence or absence of 100 ng/ml BMP-2. After the cells were harvested, the luciferase activity was determined by Top Count (Packard, USA) using a kit (Promega). The internal control plasmids were supplied by the company (Promega). The results from at least two independent experiments were evaluated.

### Quantitative real-time RT-PCR

Total RNA was extracted from cells using TRIzol reagent (Invitrogen, USA) according to the manufacturer’s instructions. The cDNA was synthesized using Superscript III reverse transcriptase (Invitrogen) according to the manufacturer’s protocol. A Light Cycler (Roche, Switzerland) and a SYBR RT-PCR kit (Toyobo, Japan) were used for quantitative real-time RT-PCR analysis in accordance with the manufacturer’s instructions. The sequences of the primer sets for the CHIP, alkaline phosphatase (ALP), Ocn and β-actin mRNAs, the target sites on the mRNAs and the PCR product sizes are shown in Table1. The relative quantification of each gene was determined using the LightCycler 480 SW software (Roche).

**Table 1:** Sequences of primers used in the real-time PCR

Genes	Primers
CHIP	F: 5′-CCTATGACCGCAAGGACATT-3′
	R: 5′-CTCTACCCAGCCGTTCTCAG-3′
ALP	F: 5′-CGGGACTGGTACTCGGATAA-3′
	R: 5′-ATTCCACGTCGGTTCTGTTC-3′
OCN	F: 5′-CTTGGTGCACACCTAGCAGA-3′
	R: 5′-CTCCCTCATGTGTTGTCCCT-3′
β-Actin	F: 5′-AGATGTGGATCAGCAAGCAG-3′
	R: 5′-GCGCAAGTTAGGTTTTGTCA-3′

F, forward primer; R, reverse primer.

### Mineralization analysis

Alizarin Red staining was used for the mineralization analysis. MC3T3-E1 cells seeded in 24-well plates were cotransfected with HA-CHIP, CHIP siRNA or empty vector. Twenty-four hours after transfection. Alkaline phosphatase staining and ALP activity was performed after the stable cells treated with TNF-α for 2 days were cultured for 21 days. Alkaline phosphatase staining and quantification were described 34. The stable cells treated with TNF-α for 2 days were cultured with or without 40 ng/ml BMP-2. After a 28-day incubation, the cells were fixed with 95% ethanol for 10 min., subjected to the Alizarin Red solution (0.2%, pH 4.5) for 10 min. and then washed twice with PBS. The Alizarin Red-S concentration was determined by measuring the absorbance at 562 nm.

### Statistical analysis

The data are presented as the mean ± SD. Comparisons were performed with Student’s *t*-test for experiments with two groups. anova was used to determine a statistical difference between multiple groups. Data from a single experiment were used to represent three independent experiments with similar results. A value of *P* < 0.05 was considered statistically significant.

## Results

### TNF-α up-regulates CHIP expression

**Figure 1:**
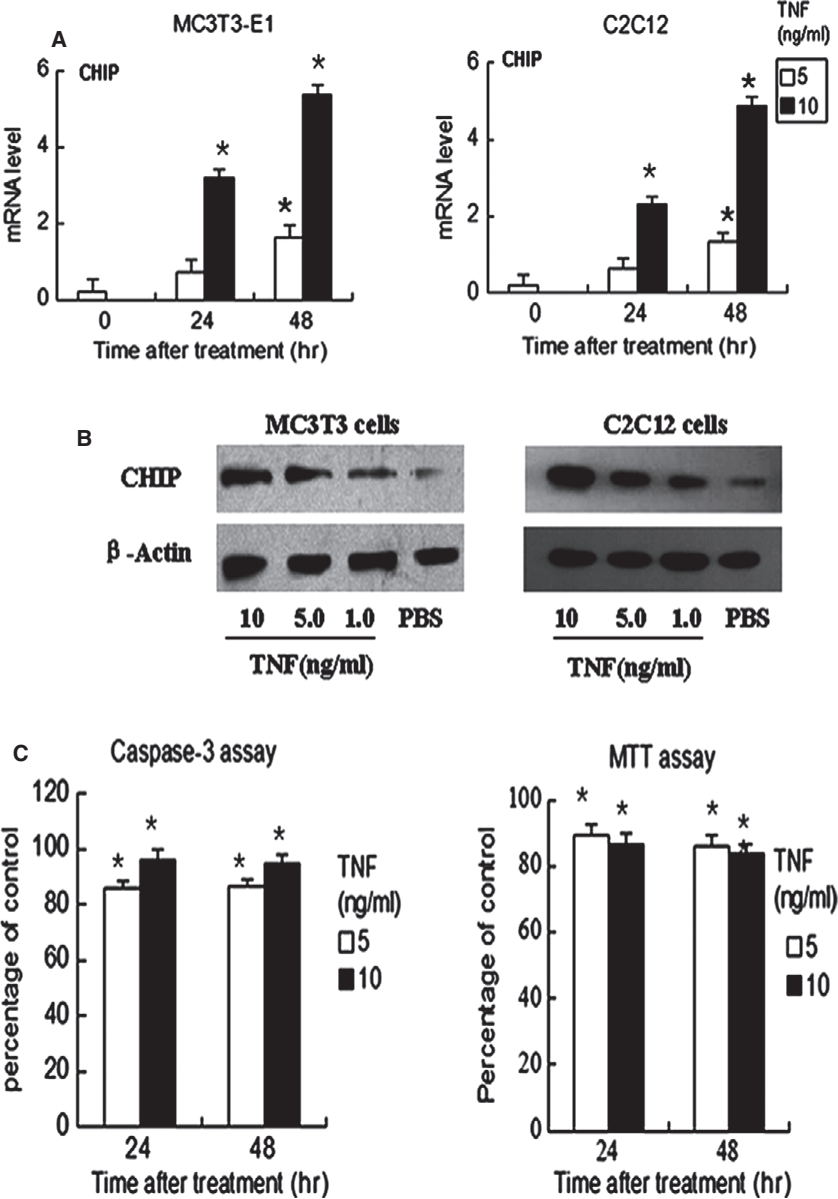
TNF-α increases CHIP expression. (A) C2C12 or MC3T3-E1 cells were cultured in medium with PBS or TNF-α (5.0–10 ng/ml) for 24 and 48 hrs. The expression of CHIP and actin mRNA was determined by real-time RT-PCR. The values are the mean ± SD of three loadings. *P < 0.05 *versus* the PBS-treated group. (B) C2C12 or MC3T3-E1 cells were treated with 5.0–10 ng/ml TNF-α for 48 hrs. CHIP and β-actin protein levels were measured by Western blot analysis. (C) Correlation between TNF-α-induced apoptosis and CHIP expression. Both cell types were treated with TNF-α (5.0–10 ng/ml) or PBS for 24 and 48 hrs, and the cell lysates were used to measure caspase-3 activity or were submitted to a MTT assay. The values are the mean ± SD of three loadings. *P < 0.05 *versus* the PBS-treated group.

Because TNF-α has been reported to enhance the expression of the E3 ligase Smurf1, we determined whether TNF-α had a similar effect on the expression of the CHIP protein. The C2C12 and MC3T3-E1 osteoblast precursor cell lines were treated with PBS or 5–10 ng/ml TNF-α, and the expression levels of the CHIP mRNA were examined by RT-PCR. The results show that the dose of TNF-α (10 ng/ml) significantly enhanced CHIP levels in both cell lines at 48 hrs (Fig.1A). Consistent with these mRNA results, TNF-α enhanced CHIP protein expression, as determined by western blot analysis at 48 hrs (Fig.1B). To confirm that up-regulated CHIP expression is associated with TNF-α-induced apoptosis, we analysed the caspase-3 activity and cell viability of both cell lines, which were treated with 10 ng/ml TNF-α for 24 and 48 hrs, using MTT (Fig.1C). At the same time, CHIP mRNA expression in the same samples was detected by RT-PCR. The results showed that the osteoblasts were morphologically normal and had normal caspase-3 activity and that CHIP expression was increased when the cells were treated with 10 ng/ml TNF-α. However, higher concentrations (>10 ng/ml) resulted in the death of the C2C12 and MC3T3-E1 cells.

### TNF-α initiates the ubiquitin pathway for the degradation of Osx

**Figure 2:**
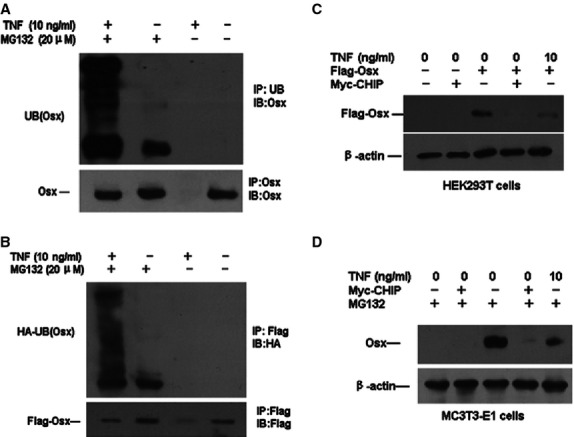
TNF-α and CHIP prompt Osx degradation through ubiquitination. HEK293T or MC3T3-E1 cells were either transfected with M-CHIP expression vector or were cotransfected with Flag-Osx expression vector or empty vector and cultured for 48 hrs in the presence of PBS or TNF-α (10 ng/ml). (A) Endogenous ubiquitinated Osx (Ub-Osx) protein ladders were detected by the anti-ubiquitin antibody (upper panel). After stripping the antibody, the total un-ubiquitinated Osx protein levels were determined using the anti-Osx antibody (lower panel). (B) Exogenous ubiquitinated Osx (Ub-HA) protein ladders were detected by the anti-HA antibody (upper panel). After stripping the blots, the total un-ubiquitinated Osx protein levels were determined with an anti-Flag antibody (lower panel). (C) The HEK293T cells were treated with PBS or TNF-α (10 ng/ml), Flag-Osx expression was examined by Western blotting with an anti-Flag antibody. (D) The MC3T3-E1 cells were treated with MG132 for the last 12 hrs. Osx expression was examined by western blotting with an anti-Osx antibody, and β-actin protein levels were determined by western blotting with an anti-actin antibody. The results are shown for one of three independent experiments.

To determine whether TNF-α promotes Osx degradation through the ubiquitin pathway, HEK293T cells transfected with the Flag-Osx and HA-UB plasmids or MC3T3-E1 cells were treated with 10 ng/ml TNF-α and the proteasome inhibitor MG132, which is an inhibitor of protein degradation through the ubiquitination pathway. Significant increases in the protein levels of both endogenous and exogenous Osx were revealed after treatment with 20 μM MG132. At the same time, we examined the Osx protein by immunoprecipitation assays using an anti-Osx antibody to precipitate the immunocomplexes and an anti-ubiquitin antibody to perform western blot analysis. This result revealed small expression of ubiquitinated Osx in vehicle-treated cells upon MG132 treatment. Interestingly, the amount of ubiquitinated Osx was significantly increased in the presence of TNF-α (Fig.2A and B). In addition, MC3T3-E1 or HEK293T cells transfected with Flag-Osx and Myc-CHIP plasmids were treated with 10 ng/ml TNF-α and 20 μM MG132, and the levels of Flag-Osx and Osx were detected by western blot analysis. As shown in Figure2C and D, 10 ng/ml of TNF-α led to a marked reduction in exogenous Osx in HEK293T cells and in endogenous Osx in MC3T3 cells. The immunoblotting results showed that the Flag-Osx protein levels decreased with increasing expressions of Myc-CHIP in the absence of TNF-α. Taken together, these results suggest that TNF-α may initiate the ubiquitin pathway to degrade Osx and that CHIP may play an important role in mediating the effects of TNF-α exposure on Osx degradation.

### TNF-α-induced effects on Osx degradation are dependent on CHIP

**Figure 3:**
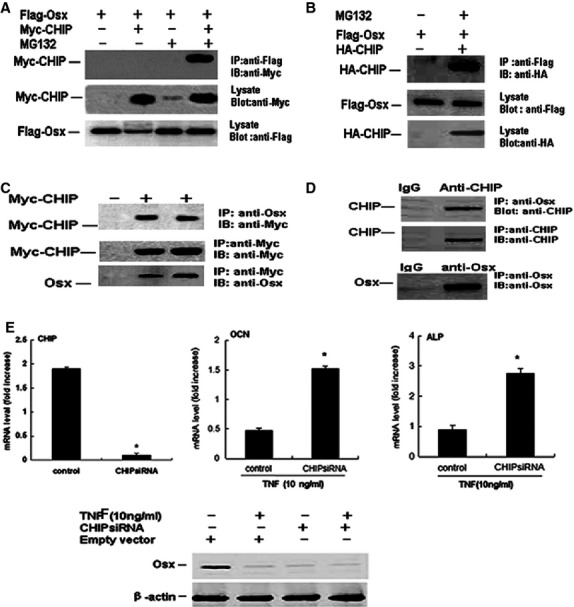
Interactions between CHIP and Osx. (A) CHIP mediates Osx degradation and ubiquitination. Flag-Osx was co-expressed with increasing amounts of Myc-CHIP in 293T cells treated with or without 20 μM MG132 for 6 hrs. Overexpressed Flag-Osx precipitated Myc-CHIP in the presence of MG132. Myc-CHIP was co-overexpressed with Flag-Osx in the absence or presence of 20 μM MG132. The precipitated band is indicated. (B) CHIP interacts with Osx. (C) Myc-CHIP interacts with endogenous Osx *in vivo* in MC3T3 cells. (D) Endogenous CHIP interacts with endogenous Osx *in vivo* in MC3T3 cells. (E) Fold increase in genes over control siRNA. MC3T3 cells were transfected with CHIP siRNA or control siRNA, and 2 days after transfection, cells were cultured in the presence with TNF-α-α (10 ng/ml) for 2 days. The expression levels of the gene of interest were examined by RT-PCR. The values are the mean ± SD of three wells. The fold increase was calculated by dividing the values of CHIP siRNA-transfected cells by the value of control siRNA-transfected cells which was normalized to 1. *P < 0.05 *versus* control siRNA. (F) Knocking down CHIP by siRNA decreases TNF-α-induced Osx degradation. MC3T3-E1 cells were transfected with vector-based siRNA targeting CHIP (CHIP RNAi) or empty vector, and the cells were then treated with 10 ng/ml TNF-α for 48 hrs. The Osx protein levels were determined by immunoblotting with an anti-Osx antibody. The values are the mean ± SD of three loadings. The fold increase was calculated as described in Figure1. *P < 0.05 *versus* the empty vector-infected TNF-α group.

To confirm that CHIP is responsible for mediating the effects of TNF-α on Osx degradation, HEK293T cells were cotransfected with Flag-Osx or Myc-CHIP expression vectors or empty vector in the absence or presence of TNF-α. The immunoblotting results showed that with increasing expression of Myc-CHIP, the Flag-Osx protein levels decreased in the absence of TNF-α. Moreover, the Flag-Osx protein levels were reduced in the presence of TNF-α (Fig.2A). Thus, we speculated that CHIP might interact with Osx and promote Osx turnover. To examine this hypothesis, we co-expressed the CHIP protein fused to a Myc tag and the Osx protein fused to a Flag tag in 293T cells using transient transfection. Forty-eight hours after transfection, the cell lysates were submitted to immunoprecipitation using an anti-Flag antibody to precipitate Osx, which showed that the CHIP protein co-immunoprecipitated with the anti-Flag antibody, as indicated by immunoblotting with an anti-Myc antibody. We could not precipitate Flag-tagged Osx co-expressed with Myc-CHIP in cell lysates in the absence of MG132. However, we observed that the Flag-tagged Osx protein precipitated the expressed Myc-tagged CHIP fusion protein in the presence of MG132 (Fig.3A). These results further reveal the interaction between CHIP and Osx in mammalian cells. Moreover, 293T cells were transfected with plasmids expressing Flag-Osx or HA-CHIP. The cell lysates were used in immunoprecipitations with anti-Flag antibody, which were then blotted with the anti-HA antibody. The expression of HA-CHIP and Flag-Osx in the cell lysates is shown in Figure3B. These data indicate that CHIP forms a complex with Osx in these mammalian cells.

Next, we tested whether Myc-CHIP could interact with endogenous Osx. We specifically precipitated endogenous Osx from MC3T3-E1 cells treated with Myc-CHIP using an anti-Osx antibody. Interestingly, the Myc-CHIP protein was reprecipitated in the precipitated complex containing endogenous Osx (Fig.3C). At the same time, we further demonstrated that endogenous CHIP and Osx were associated and that endogenous CHIP could form a complex with endogenous Osx (Fig.3D). The above results reveal that CHIP indeed interacts with Osx and that Osx may be a substrate of CHIP-mediated ubiquitination.

To further investigate the role of CHIP in TNF-α-induced Osx degradation, MC3T3-E1 cells, in which endogenous CHIP is moderately expressed, were infected with a retroviral supernatant containing siRNA specific for CHIP to knock down endogenous CHIP in the presence of TNF-α. Then, the expression of osteoblast differentiation marker genes (ALP and OCN) was detected by real-time RT-PCR. This treatment resulted in a significant increase in the expression of Osx protein in the cells. CHIP siRNA decreased TNF-α-induced CHIP expression and led to increase the ALP and OCN expression (Fig.3E) and TNF-α-induced inhibition of Osx degradation (Fig.3F). The expression of these genes was decreased in the overexpression clones and enhanced in the depletion clones, further suggesting that the TNF-α-induced effects on Osx degradation may depend on CHIP and that the ubiquitin pathway is involved in regulating Osx. Thus, identifying the Ub Lys acceptor site(s) of Osx and determining the functional effects of removing these site(s) on Osx activity are interesting.

### The residues K55 and K386 of Osx are involved in ubiquitination

**Figure 4:**
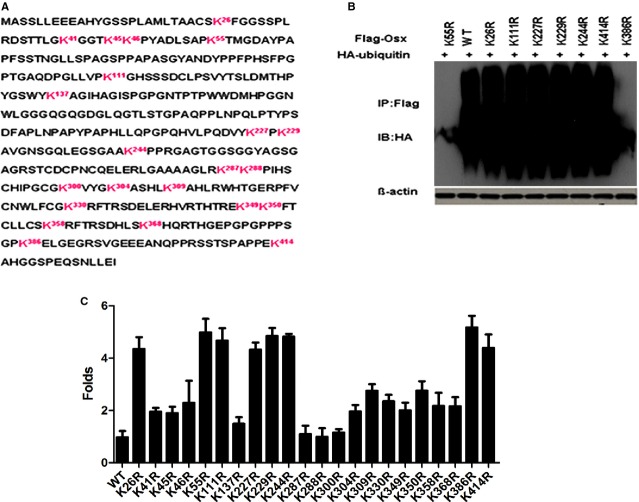
The residues K55 and K386 are the ubiquitination sites of Osx. (A) Mouse Osx primary sequence with the lysine residues indicated. (B) HEK 293T cells were cotransfected with HA-Ub expression plasmids and the WT Flag-Osx plasmid or the K26R, K55R, K111R, K227R, K229R, K244R, K386R or K414R mutants. Cell lysates were immunoprecipitated using an anti-Flag antibody and blotted with an anti-HA antibody. The results are shown for one of three independent experiments. (C) HEK 293T cells were cotransfected with the Ocn-Luc reporter, control *Renilla* luciferase reporter vector, and the WT Flag-Osx plasmid or Lys-to-Arg mutants. The relative luciferase activity was measured 24 hrs after transfection. The results were obtained from three independent experiments performed in triplicate. The data are expressed as the mean ± SD.

Because the mutation of Lys residues may induce structural changes in the Osx protein and also affect its activity by blocking ubiquitination, each of the Lys residues of Osx was replaced with an Arg residue (Fig.4A), which maintained the positive charge but could not serve as an acceptor site for ubiquitination. To identify the ubiquitination sites in Osx, Osx-deficient HEK293T cells were cotransfected with the WT Flag-Osx plasmid, mutants with different Lys-to-Arg mutations and Ocn-Luc constructs. Overexpression of the K26R, K55R, K111R, K227R, K229R, K244R, K386R and K414R mutants increased Ocn-dependent luciferase reporter gene expression by more than 2.6-fold, compared with WT Osx. Eight of 22 Lys residues in Osx were able to accept the ubiquitin molecule (Fig.4C), and the Lys-to-Arg mutations at these sites might abrogate Osx ubiquitination and degradation. To further analyse the Ub Lys site(s), we cotransfected the WT Flag-Osx or the eight above-described Lys-to-Arg mutants, along with a HA-ubiquitin vector, into HEK293T cells. We found that only the K55R and K386R mutants displayed dramatically decreased ubiquitiation. We demonstrated that K55 and K386 are the ubiquitination sites of Osx by co-immunoprecipitation assays. These results indicated that the Osx K55R and K386R mutations reduced Osx ubiquitination activity to a level comparable with the result obtained with the wild-type control (Fig.4B), suggesting that these two residues may be ubiquitination sites of Osx.

### CHIP mediates the inhibition of BMP Signalling and Osx by TNF-α through the K55 and K386 UB sites of Osx

**Figure 5:**
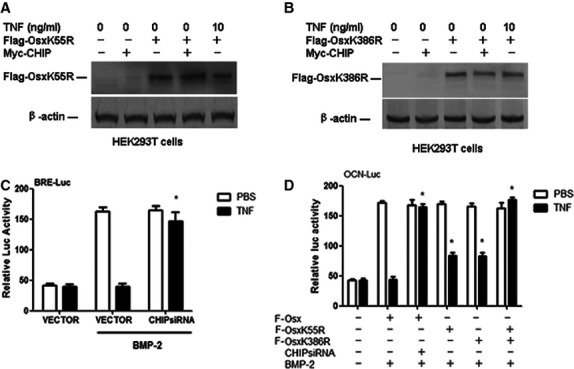
CHIP mediates the inhibition of BMP signalling and Osx by TNF-α through the K55 and K386 UB sites of Osx. (A and B) MC3T3-E1 cells were transfected with vector-based siRNA targeting CHIP (CHIPRNAi) or empty vector. The cells were then transfected with the Flag-Osx expression plasmid and Osx reporter vector (OC-Luc) or BMP reporter vector (BRE-Luc) for 6 hrs, treated with 10 ng/ml TNF-α for 48 hrs and then stimulated with 40 ng/ml BMP-2. Then, the cell lysates were extracted, and luciferase activity was measured using a Dual Luciferase Reporter Assay System. The values are the mean ± SD of three loadings. *P < 0.05 *versus* the empty vector-infected TNF-α group.

CHIP has been reported to be an E3 ubiquitin ligase that regulates BMP signals by promoting the ubiquitination and proteasome-dependent degradation of Smad1/5 35. Our above results show that TNF-α decreases Osx expression through the up-regulation of CHIP. To further confirm that TNF-α mediates the inhibition of Osx expression by CHIP through the K55 and K386 UB sites of Osx, HEK293T cells transfected with the Flag-Osx, Myc-CHIP, K55R and K386R mutant plasmids were treated with 10 ng/ml TNF-α, and the levels of K55R and K386R were detected by Western blot analysis. The immunoblotting results showed that with increasing expression of Myc-CHIP, the K55R and K386R protein levels showed little decrease in the absence or presence of TNF-α (Fig.5A and B), suggesting that TNF-α mediates the inhibition of Osx expression by CHIP through the K55 and K386 UB sites of Osx. Furthermore, we examined whether TNF-α targets BMP signalling. MC3T3-E1 cells transfected with the OCN-Luc reporter, Flag-Osx vector, K55R and K386R mutants and the BRE-Luc reporter, which is a BMP-specific transcription reporter driven by the BMP-responsive elements of the *Id1* gene, were stimulated by BMP-2 in the presence or absence of TNF-α in a luciferase reporter assay. We tested the role of CHIP as a mediator of the TNF-α inhibition of Osx by silencing CHIP with a short interfering RNA (siRNA). The results indicated that CHIP dramatically inhibited the Osx-induced transcriptional activity increase that was observed in the Luciferase reporter assay in MC3T3-E1 cells stimulated with BMP-2 or TNF-α. CHIP siRNA reduced the inhibitory effect of TNF-α. Furthermore, when the endogenous CHIP protein was depleted in MC3T3-E1 cells by CHIP siRNA, we observed an increase in the luciferase activity at basal expression (without the overexpression of Osx) or under Osx overexpression. These data suggest that CHIP siRNA decreases the effect of TNF-α on the Osx reporter. However, we observed that CHIP depletion by siRNA reduced TNF-α-induced inhibition of the BMP-2 signalling reporter (Fig.5C). The K55R and K386R mutants decreased the effect of CHIP on the Osx reporter in the presence of TNF-α (Fig.5D). Thus, although TNF-α inhibits BMP signalling through CHIP, its inhibition of Osx is mediated by CHIP through the K55 and K386 sites.

### TNF-α affects osteoblast differentiation and mineralization of MC3T3-E1 cells through CHIP

**Figure 6:**
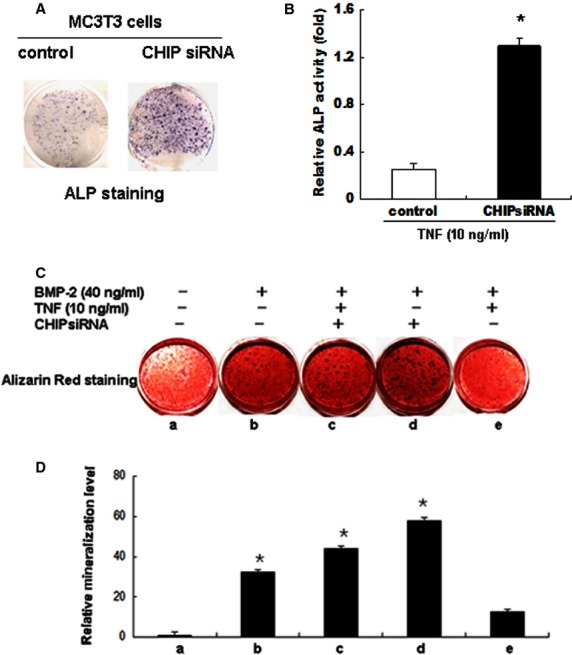
TNF-α decreases osteoblast mineralization *via* CHIP. Establishment of cell lines stably expressing HA-CHIP in MC3T3-E1 cells. (A) The stable cells transduced with adenoviruses expressing CHIP siRNA were subjected to ALP staining. (B) The ALP activity of the stable cells was measured after 21 days of differentiation in the absence or presence of TNF-α. (C) The stable cells were transduced with adenoviruses expressing CHIP siRNA or the empty virus for 6 hrs, treated with 10 ng/ml TNF-α for 48 hrs and then stimulated with 40 ng/ml BMP-2 for 28 days followed by the determination of mineralization by Alizarin Red staining. Images of mineralization by Alizarin Red staining of three independent experiments. (D) Quantification of the mineralization. The results are the mean ± SD of three loadings. *P < 0.05 *versus* empty vector-infected TNF-α group.

To further evaluate the potential roles of CHIP on osteoblast function under conditions in which TNF-α is overexpressed, we established stable MC3T3-E1 cell lines overexpressing HA-CHIP or depleted of endogenous CHIP by siRNA. After the stable cell lines treated with TNF-α for 2 days were cultured for 21 days, ALP staining and ALP activity was performed (Fig.6A and B). At 28 days, the stable cell lines were stained with Alizarin Red to identify mineralized nodules. In the absence of TNF-α, a 40 ng/ml dose of BMP-2 enhanced the Osx protein levels and number of nodules in CHIP knockdown clones compared with the HA-CHIP-expressing clones. Similarly, the Osx protein levels and number of nodules increased in the CHIP knockdown clones compared with the controls. However, in the presence of TNF-α, there was an apparent TNF-α-induced decrease in the Osx protein levels and in mineralized nodule formation in the HA-CHIP-expressing clones compared with the CHIP knockdown clones (Fig.6C and D). These data showed that CHIP decreased the number and size of the mineralized nodules under normal and inflammatory conditions, suggesting that CHIP overexpression prevents or delays cell differentiation into mature osteoblasts and that the depletion of CHIP promotes or enhances this process.

## Discussion

Bone destruction is marked in RA, a disease characterized by proliferative synovitis in which proteases secreted by the synovial membrane cause cartilaginous inflammation that leads to joint destruction 36. Tumour necrosis factor-α is the key mediator of joint inflammation and bone destruction in inflammatory arthritis, *e.g*. RA and AS. Several studies have detected high amounts of TNF-α in the serum and synovial fluid of patients with RA and AS 37,38, but the molecular mechanisms that mediate the inhibitory effects of TNF-α on osteoblasts have not been fully examined. Here, we provide evidence for the regulation of the stability of the osteoblast-specific transcription factor Osx by TNF-α through the E3 ligase CHIP. Our findings provide the first evidence that TNF-α inhibits osteoblast function by controlling the ubiquitination status of the Osx protein through CHIP. Furthermore, we believe that this is the first demonstration showing that the K55 and K386 residues are the ubiquitination sites of mouse Osx, and that TNF-α affects osteoblast differentiation and mineralization through CHIP.

Runx2 and Osx are critical transactivators of osteoblast differentiation, and the activities of these two transcription factors are tightly regulated at different levels, including transcription, translation, phosphorylation, acetylation, protein–protein interactions and ubiquitin-mediated degradation 39–42. Previously, several other E3 ligases, including CHIP, which is an E3 ubiquitin ligase, were reported to regulate Runx2 protein turnover. CHIP has been reported to directly interact with Runx2 and to induce the ubiquitin-dependent degradation of the Runx2 protein 43,44. These findings indicate that CHIP-mediated protein degradation plays a role in osteoblast differentiation. However, Osx is essential for the expression of many bone-related genes. Thus, the characterization of the role of the Osx transcription factor in regulating bone-related genes is important. Currently, the regulation of Osx has not been fully investigated, and knowledge of the post-translational regulation of Osx is especially sparse.

Ubiquitination is a post-translational modification that regulates the function, localization and turnover of cellular proteins to impact cellular morphology, activity and interactions within multicellular organisms. CHIP is a cochaperone protein identified through its interaction with Hsc/Hsp70, and promotes the ubiquitination and degradation of chaperone-bound proteins. It has been reported that CHIP expression is gradually decreased during osteoblast differentiation but maintained at a high level in non-osteoblast lineages 45. In this study, we show that CHIP is a novel post-translational regulator of Osx through several lines of evidence, and our data suggest that CHIP may play an important role in the regulation of Osx protein levels in osteoblast precursor cells upon treatment with TNF-α.

As CHIP affects protein degradation through the ubiquitination process, we examined whether CHIP is involved in Osx ubiquitination. These experiments demonstrated that Osx indeed interacted with CHIP. Whether there is an adaptor involved in the regulation of the CHIP-Osx interaction remains to be determined. In an experiment with the Osx mutants, we screened for potential ubiquitination sites in mouse Osx by testing the Lys-to-Arg mutant plasmids and luciferase reporter assays. We found that the K55R and K386R mutants induced markedly decreased polyubiquitination, suggesting that these two residues might be the ubiquitination sites of Osx. To further confirm that TNF-α mediated the inhibition of Osx expression by CHIP through the K55 and K386 UB sites of Osx, Our results show that the interaction between Flag-Osx (K55R and K386R) and Myc-CHIP was barely detectable after a short exposure and was still only weak after a long exposure and that WT Flag-Osx interacts strongly with Myc-CHIP. Furthermore, we knocked down endogenous CHIP expression using siRNA in the MC3T3-E1 cell line upon TNF-α treatment and found a significant increase in the amount of exogenous and endogenous Osx protein, suggesting that the TNF-α-induced effects on Osx degradation depended on CHIP.

The intracellular signalling of BMP is mediated primarily by the Osx protein, an essential transactivator of osteoblast differentiation and osteoblast differentiation. We questioned whether CHIP overexpression would also block these signals in osteoblasts. We observed that CHIP depletion by siRNA reduced the TNF-α-induced inhibition of the BMP-2 signalling reporter. The K55R and K386R mutants decreased the effect of CHIP on the Osx reporter in the presence of TNF-α. Thus, although TNF-α inhibits BMP signalling through CHIP, its inhibition on Osx is mediated by CHIP through the K55 and K386 sites.

We established stable MC3T3-E1 cell lines overexpressing HA-CHIP or depleted of endogenous CHIP by CHIP siRNA. The stable cell lines were treated with BMP-2 or TNF-α and analysed for Osx expression. The results showed that CHIP decreased the Osx protein levels in MC3T3-E1 cells. Our data suggest that CHIP is a key regulator in the TNF-α-mediated inhibition of osteoblast differentiation and mineralization.

On the basis of these findings, we propose a model to explain the involvement of CHIP in inhibition of osteoblast differentiation in patients with chronic inflammatory disorders. In arthritic joints, secretion of pro-inflammatory cytokines, such as TNF-α, is elevated. Tumour necrosis factor-α can affect osteoblasts locally or at distant sites to increase their expression of CHIP. CHIP promotes the ubiquitination degradation of Osx proteins, resulting in consistently decreased steady-state levels of these key regulators in osteoblasts. Consequently, osteoblast differentiation cannot match the increased bone resorption, leading to systemic bone loss.

Although our data indicate that CHIP may be the primary mediator of TNF-α-induced degradation of Osx, it does not mean that CHIP is the only mechanism by which TNF-α inhibits osteoblast function. It has become evident recently that ubiquitin protein ligases can be regulated by other mechanisms, including phosphorylation of the ligase or substrate, utilization of adaptor proteins or intra- and inter-molecular interactions 46–48. Whether or not these regulatory mechanisms contribute to increased degradation of Osx proteins in TNF-α-overexpression condition is not known. These possibilities need to be investigated in the future. Thus, TNF-α mediated osteoblast inhibition likely works through multiple mechanisms. In the future, it will be interesting to determine if post-translational modifications mediated by CHIP influences key signalling pathways in osteoblasts.

Collectively, in this study, our data have uncovered a novel aspect of the molecular mechanism that mediates the effect of TNF-α on osteoblast inhibition. We have identified, for the first time, CHIP E3 ligase as a molecular mediator of the TNF-α inhibition of osteoblast differentiation through Osx ubiquitination and protein degradation. Moreover, we identified that the K55 and K386 residues are the ubiquitination sites of Osx and demonstrated that the ubiquitination of Osx plays a key role in osteoblast differentiation. These findings enrich our knowledge of the role of TNF-α in the proteasomal regulation of protein function by CHIP E3 ligase in osteoblasts. Thus, these results may have implications in targeting CHIP as a therapeutic strategy for treating inflammatory bone loss.

## Funding

This work was supported by grants from the National Natural Science Foundation of China (30600560). A Project Funded by the Peak Foundation Six Talent of Jiangsu Province of China. The funders had no role in study design, data collection and analysis, decision to publish or preparation of the manuscript.

## Conflicts of interest

The authors confirm that there were no conflicts of interest.

## Author contribution

JMX participated in the experiments designs, collecting data, statistical analysis, manuscript drafting and correspondence to editor. JRG participated in experimental conception and critical reading. All authors read and approved the final manuscript.
